# Teachers’ views on disinformation and media literacy supported by a tool designed for professional fact-checkers: perspectives from France, Romania, Spain and Sweden

**DOI:** 10.1007/s43545-022-00340-9

**Published:** 2022-04-09

**Authors:** Thomas Nygren, Divina Frau-Meigs, Nicoleta Corbu, Sonia Santoveña-Casal

**Affiliations:** 1 Department of Education, Uppsala University, Uppsala, Sweden; 2 Sorbonne Nouvelle University, Paris, France; 3 National University of Political Studies and Public Administration, Bucharest, Romania; 4 Universidad Nacional de Educación a Distancia (UNED), Madrid, Spain

**Keywords:** Disinformation, Media and information literacy, Fact-checking tools, Teaching and learning, Transliteracy, Technocognition

## Abstract

The current media eco-system has become more and more polluted by the various avatars of “fake news”. This buzz term has been widely used by academics, experts, teachers and ordinary people, in an attempt to understand and address the phenomenon of information disorder in the new media environment. However, studies have rarely questioned what teachers, key stakeholders in the media literacy field, actually understand by “fake news”, and to what extent the new digital tools available to fact-check are actually viable solutions to fight disinformation actively. In this context, we conducted focus groups (*N* = 34 people interviewed in 4 focus groups) with teachers in four countries (France, Romania, Spain and Sweden), in order to assess their understanding of “fake news”, as well as their perception of possible measures to combat the phenomenon, with a particular focus on digital tools. The findings show that the understanding of the concept of “fake news” differs from one country to the other, but also within the same country, with a common feature across countries: intention to deceive. Additionally, respondents identified lack of media and information literacy (MIL) in education as a major gap for combatting information disorders. Furthermore, they find that the use of digital tool for professional fact-checking needs to be repurposed or followed by pedagogical instructions to fit into the complexity of educational practices. Our findings highlight possible solutions for MIL in education using a combination of technocognition and transliteracy as theoretical framework and scaffolded pedagogical design for better adoption of fact-checking techniques.

**Supplementary Information:**

The online version contains supplementary material available at 10.1007/s43545-022-00340-9.

## Introduction

Disinformation, in the shape of ‘fake news’, has attracted public attention on the dual needs for fact-checking and for media literacy (Frau-Meigs 2019). A number of fact-checking initiatives and tools have emerged as a response from the media professionals. Such tools are mostly geared towards journalists, not towards teachers, students or citizens at large. Research reveal additional gaps: the focus is mostly on text-based ‘fake news’, much less on visual ‘fake news’, though these are among the most prominent in social media; building resilience implies to navigate online information in new ways, as professional do fact-checkers. For this, people seem to need a mix of content knowledge, constructive attitudes and digital skills as highlighted by theories of media literacy as transliteracy (Frau-Meigs 2013) and technocognition (Lewandowsky et al. 2017).

Faced with challenges of information disorder and infodemics, there is a loud call for educational actions to support citizens when flooded by information of various quality in multimodal formats (World Health Organization 2020; European Commission 2018). Scholars note that digital media may support but also pose a serious threat to democracy in different countries (Lorenz-Spreen et al. 2021). The importance of learning how to think and act in a world of disinformation is underscored (Kozyreva et al. 2020). Education is described as key since automated fact-checking holds important limitations, not least when it comes to debunking visual images and deep fakes (García Lozano et al. 2020; Neekhara et al. 2020). Disinformation, defined as inaccurate, manipulative or falsified information that is deliberately designed to mislead people, is hard to detect. So is its amplification that can be powered by all sorts of stakeholders, young people included, and arguably explains the perceived novelty and virality of the ‘fake news’ phenomenon. Not least since disinformation is often a mix of credible, biased and false information, or a selection of facts presented to support a false narrative. Education is necessary to deal with this complicated issue, but education is not a quick fix (Nygren 2019), and implementing digital tools to support citizens may hold special challenges. Introducing a professional tool for fact-checking in education may have little effect if the tool is not understood or not found useful by teachers and students. Therefore, it is central to understand better how teachers view the problems of disinformation and perceive how a digital tool designed to fact-check multimodal information may be helpful in education. Additionally, considering how context matters for teaching and learning, we find it important to gather teachers’ perceptions across cultural borders to support a design process that rises to the challenge of the cross-national nature of the Internet (Frau-Meigs 2019), as its contents are not hindered by natural frontiers or national sovereignty.

The purpose of this study is to better understand potential possibilities and challenges of using a new digital tool to fight disinformation in education across national and cultural borders. To understand different contexts, we study teachers’ views on topics related to disinformation and we investigate three nested sub-questions:

RQ1.: What are teachers’ own patterns of news consumption?
RQ2.: What do teacher understand by the term ‘fake news’?
RQ3.: What are, in teachers’ view, the most effective ways (measures to be taken) to fight disinformation (MIL included)?

Against this backdrop, we also investigate the main research question of this study, namely:

RQ4.: How do teachers perceive the introduction of a digital tool (InVID-WeVerify) to address the challenge of disinformation in classrooms across national and cultural borders?

To better understand the possible potentials and pitfalls of introducing new tools against disinformation, we asked teachers in France, Romania, Spain and Sweden to test and reflect upon the usefulness of a digital fact-checking tool, InVID-WeVerify, in teaching.

InVID-WeVerify is a free verification plug-in, downloaded more than 40,000 times, used across the globe by professional journalists and fact-checkers to verify images and videos. It is available in multiple languages and used in newsrooms such as France24, India Today, Canal 1 and Volkskrant (Bontcheva 2020). For instance, fact-checkers at Agence France-Presse (AFP) use it on a daily basis to verify rumours and suspicious content on COVID-19 or US elections. It offers several functionalities, like image similarity, image reverse search, metadata analysis, video keyframes automated extraction and image forensics. In this study, we analyse teachers’ responses in relation to their views on disinformation in society and schooling, in the light of theories of media literacy, after they have been introduced to this digital fact-checking tool. A first set of questions dealt with their understanding of the ‘fake news’ concept, perceived incidence of the phenomenon, possible effects, and prior knowledge and use of similar tools. A second set of questions was focused on the fact-checking tool itself (see Appendix 1).

To process and interpret the results, we drew on a combination of theories from media and information literacy (MIL), technocognition, and education. MIL, as “transliteracy” (Frau-Meigs 2013), was used to articulate the multi-media dimension of fake news (across mass and social media) and the trans-domain convergence of information as computation (data), communication (media) and curation (documents). Transliteracy in MIL combines the agency and autonomy of the user with the capacity to navigate and discriminate online sources, especially when dealing with (meta)data and media. Transliteracy, defined as ‘the ability to read, write and interact across a range of platforms, tools and media’ (Thomas et al. 2007), tends to focus on interactions between users and screens. As such, it ties to theories of technocognition highlighting the importance of focusing on updated design of information architectures and education to support cognition against the spread of disinformation. Advocates of technocognition highlight how journalistic principles and technology need to go hand in hand to educate and support citizens and safeguard democracy (Lewandowsky et al. 2017). Additionally, education theories about curricular processes were brought in to understand teachers’ perceptions of their scope of action in ‘the arena of realisations’, in relation to formal guidelines from “the arena of formulations” (Lundgren 1990) and surrounding society (Goodlad 1979), also discussed below. Theories presented by Goodlad (1979) and Klafki (1995) underpin how teaching and learning are complex matters, where teachers’ reflections from practice are central. These approaches converge in their emphasis on the need for teacher agency, and their legitimacy in identifying what needs to be done and how (Lindensjö and Lundgren 2000). Using this combination of theories our aim is to contribute to the research field of MIL and our findings will be of interest to researchers, decision makers and teachers interested in implementing digital tools to combat disinformation in classrooms across cultural borders. Similarities and differences identified across national borders may inform designers, teachers and decisionmakers about how to address some of the challenges in the complex process of supporting education against disinformation in different educational settings.

## Previous research

The importance of MIL has been emphasised by scholars in theory and practice (Kahne and Bowyer 2017; Frau-Meigs et al. 2017; Mihailidis 2018). MIL has surfaced in public policy decisions all over Europe, especially spurred by the 2015 wave of terrorist attacks, followed by the 2016 wave of ‘fake news’, where propaganda and other information troubles lead politicians to realise the threat to democracy posed by lack of MIL. Consequently, MIL has become an indispensable part of the new public policies, introduced and aligned with the democratic standards of the EU. Specifically, the European Directive of Audiovisual Media Services, of 2018, makes it an obligation for states to implement MIL and digital platforms to support it. However, public policies differ vastly across Europe, from school curricula and resources that fully incorporate MIL (France, Sweden) to others that tend to consider it as a second curriculum (Romania, Spain) and leave it to non-school actors in civil society or the private sector.

In relation to the arena of formulations, the approach of Hallin and Mancini can cast light on the media literacy situation. According to Hallin and Mancini (2011), there are four main models for media policy: ‘the Polarised pluralist model’, characteristic of the Mediterranean countries (including, France and Spain); the ‘Democratic corporatist model’, characteristic of the North/Central European countries (i.e. Sweden); the ‘Liberal model’ that is being developed in the North Atlantic countries, to which a fourth model, which is the ‘Hybrid model’ of post-communist countries has been added (Romania is a typical example). The East-European countries are arguably hybrid models, as they have experienced recent political and institutional reforms from the authoritarian to the multi-party parliamentary system, free elections and the rule of law, as well as liberalisation of economy. The four dimensions, across which Hallin and Mancini (2011) built their theory, are related to the structure of the media market, political parallelism (that is, how much political oriented media are in each country), professionalisation of the journalistic field, and the role of the state.

Matović et al. (2017) have added a fifth dimension, MIL, to Hallin and Mancini’s model. The additional criteria they consider for the validity of this fifth dimension are: the level of professionalisation of teachers, the degree of state intervention, the presence of MIL in the school curricula, and the role of actors outside schools (civil society) as well as the presence and acceptance of information and communications technology in schools. This fifth dimension thus incorporates the arena of formulations and the arena of realisations, to provide country profiles for media literacy implementation.

Research on teachers’ views, affecting the arena of realisations, is less considered; yet, they have to incorporate the multiple levels of interplay between school managers, teachers and society at large (especially parents and families, as disinformation seems to be partly embedded at home). Teachers’ views on news may affect their teaching, and what teachers do in the classroom may definitely impact students’ abilities to navigate misinformation in updated ways (McGrew 2020; McGrew et al. 2019). However, very interested teachers may also still struggle to teach students to evaluate digital news (McGrew 2020). Teaching students to determine credibility of digital news has been noted to hold multiple challenges, not least students’ lack of MIL (Nygren et al. 2020).

This problem can be linked to notions about how hard it is to navigate information in a post-truth era (Lewandowsky et al. 2017; Wineburg and McGrew 2019). Not least, young people, growing up in a digital era, have a hard time separating credible news from misleading information (Breakstone et al. 2019; Ku et al. 2019; McGrew et al. 2018; Nygren and Guath 2019, 2021). Navigation in clever ways needs to be supported by digital resources. Experts determining credibility of news use digital tools to aid them, which could also be a benefit for citizens.

Thus, in the light of previous research, it is evident that citizens need cognitive abilities adapted to technology to navigate information in an era of disinformation (Lewandowsky et al. 2017; Rich 2018). However, previous research has not investigated the extent to which digital tools can be used to support teachers and students. Previous research has, nonetheless, noted that teachers’ views are essential when implementing new technology in classrooms (Heath 2017; Sugar et al. 2004). Teachers may have different priorities which impact how and if they use digital tools to support learning (Cuban et al. 2016; Kurt 2012). Noting how teachers’ perspectives are key when implementing new technology in classrooms and how their perceptions of news credibility may influence their teaching, we find it is central to gather and analyse their views on news and disinformation, and potentials and pitfalls with using a tool for professional fact-checkers in classrooms. Taking the various European models for policy integration (Polarised pluralist, Democratic corporatist, and Hybrid) into account may also enable comparisons across cultures and point to alternative cross-national solutions.

## Digital tools in teaching

Today, it is possible for almost anyone to produce and share texts, images and videos, which are hard to be fact-checked for people and machines (Kim et al. 2018; Neekhara et al. 2020). New media and digital journalism can support democracy, but at the same time they facilitate the spread of exaggerations and lies in various ways (Del Vicario et al. 2016; Guess et al. 2019; Vosoughi et al. 2018). Noting how in many cases it is not possible for people to separate a fake video from a credible video calls for a combined approach where MIL develops critical thinking with the support of a technological artefact that is not just a simple tool. Specifically, MIL tends to question the strictly operational and instrumental approach to education, preferring devices to be nested in sense-making practices and tasks (Frau-Meigs 2019; Nygren 2019).

Transliteracy accommodates this tool entry in the case of InVID-WeVerify as the plug-in has embedded support for cognitive processes in its multiple functionalities. InVID-WeVerify is a plug-in designed to help journalists and news professionals in their efforts to debunk fake news and verify user-generated videos and images (InVID-WeVerify 2019; Teyssou et al. 2017). It is designed to make it possible to: (a) retrieve metadata about videos and images, (b) fragment videos into keyframes to allow image-similarity search in other contexts, (c) perform advanced search queries on Twitter, Facebook and YouTube, (d) compare the efficiency of search engines (Google, Yandex, Baidu…), (e) look inside images through a magnifying lens and (f) analyse an image with forensic filters (to detect alterations within its structure such as quantisation, frequencies, colours, pixel coherence). All these itemised functionalities correspond to cognitive processes such as retrieve, fragment, search laterally, compare across data sets and apply filters. Using digital tools when corroborating information is emphasised in theories of technocognition (Lewandowsky et al. 2017). Researchers underscore the high potential for developing updated digital heuristics, among teachers and students (McGrew et al. 2017).

In design-based research, it has been noted how it is essential, and also a research challenge, to have a dialogue with teachers about information and communication technology in education when trying to solve real-world problems (Akkerman et al. 2013). Thus, new technology needs to be carefully implemented with greater attention paid to teachers’ views and to their interactions with digital artefacts. This approach brings together the potential of transliteracy in dialogue with technocognition as a theoretical framework for MIL.

## Methodology: design-based research

With inspiration from design-based research, we find it important to study how teachers perceive the so-called fake news phenomenon, and the InVID-WeVerify tool in the light of their experiences. In line with most design-based research, we investigate the possibilities for educational improvement with an aim to develop new methods and materials, useful in the complexity of everyday classroom practices (Anderson and Shattuck 2012; Kelly 2004). Design-based research has been criticised for being a top-down process, where interests of researchers are prioritised over those of teachers and students (Engeström 2008). To address this issue, we include teachers in the design process and discuss their views in this paper. We also designed a ‘transliterate situation’, as we asked them to test the tool and to respond to their interactions with the tool with an accompanying lesson plan on disinformation and attendant activities, implying the trans-domain areas of curation (documents), computation (data) and communication (media). We expected to see their interactions and constraints as they had to deal with a new device, where each cognitive act is affected by the level of agency and finality of the specific activity (Delamotte et al. 2014).

Interviewing teachers and having them test the InVID-WeVerify tool makes it possible for us to pay attention to their wisdom of practice (Shulman 2004) and their technological pedagogical content knowledge (Koehler and Mishra 2009). Understanding professional teachers’ perspectives on disinformation and the digital tool for fact-checking can provide us with important insights in the process of working collegially, with the aim to ‘test and build theories of teaching and learning, and produce instructional tools that survive the challenges of everyday practice’ (Shavelson et al. 2003, p. 25). Testing and developing digital tools that may hold new dimensions and practices is often at the core of design-based research (Anderson and Shattuck 2012), not least since this may provide new theoretical insights.

This research is part of the Youcheck! Project, funded by the European Union (2019–2020 within its call “media education for all” (www.project-youcheck.com) and in this paper, we present an early and crucial stage in a design process moving towards classroom interventions. The meetings with teachers will be followed by design iterations, where the methods and materials will be further developed in collaborative processes, tested in classroom interventions and evaluated for further developments. Finally, if found useful in the complexity of practice, they will be shared with teachers with open-access, within the framework of the Youcheck! project. However, this all starts (and ends) with the key actors in education, the teachers. We are thus aligned with previous call for ‘research that features practitioner co-creation of knowledge as a vehicle for use and uptake’ (Ormel et al. 2012, p. 98).

### The complexity of implementation

An analytical framework, inspired by Goodlad (1979) and his description of implementation as a matter of multiple levels of curricula in constant interplay with each other and society at large, guided our meetings with teachers. We perceive implementation of international guidelines [such as the Audiovisual Media Services Directive (AVMS) in the case of media education] as far more than just a top-down process (Nygren 2016). It is a matter of interaction with transactions, interpretations or the lack thereof. International ideas formulated in multiple ideological curricula may be included in formal curricula in different countries and implemented in the classrooms by teachers in diverse ways, or not all. Bottom-up influences are also part of this process, previously described as two levels of curriculum: one where the guidelines are formulated (arena of formulations) and one where teaching is carried out (arena of realisation) (Lundgren 1990). It is important to note in detail what teachers see as relevant regarding contents, methods and goals of education in reforms formulated, since they are central on the level where teaching and learning happens in schools. In parallel, it is important that teachers are given legitimacy in identifying what needs to be and what can be done (Lindensjö and Lundgren 2000).

**Fig. 1:**
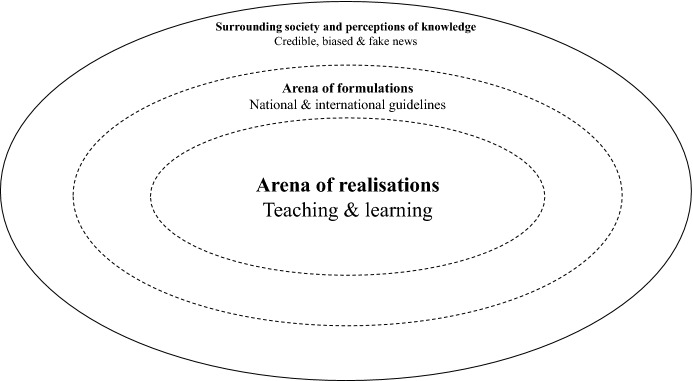
Analytical framework of the study regarding implementation with inspiration from Goodlad (1979), Klafki (1995) and Lundgren (1990)

Accordingly, we opted for a qualitative methodology, using focus groups, as we were looking for in-depth understanding of the phenomenon under study. Many studies approaching similar topics focus on more quantitative designs, but especially in this comparative setting, in which contrasting and comparing patterns of behaviour was essential, we opted for a more qualitative approach. Therefore, we decided to engage teachers to get their perspectives on disinformation in society, their views on the guidelines they receive for MIL and whether a tool for debunking fake news may be used in education to support students in the arena of realisations (see Fig. 1).

Figure 1 shows how teaching and learning in the arena of realisations are conducted in relation to the arena of formulations (Lundgren 1990) and perceptions of knowledge in society (Goodlad 1979). In line with Klafki (1995), we note teachers’ reflections from teaching as important and put this in the centre. The blurry lines between the arenas in Fig. 1 symbolise boundaries of transactions, interpretations and barriers between the arenas and the surrounding society. Bearing in mind the central role teachers play to make learning happen, we focus on the arena of realisations and our questions in the focus groups place teachers’ reflections in relation to national and international formulations about education against disinformation and teachers’ views on disinformation in society.

### Sample

To safeguard a variety of input in the process, we decided to get contributions from teachers in very different settings. We used convenience sampling in four countries and all participants volunteered to participate in focus groups about misinformation and media literacy. We acknowledge that participants may be more interested in media literacy than other teachers may be since they agreed to participate. Participants in Romania (*N* = 9) were all teachers in upper-secondary schools across Bucharest, teaching history, geography, psychology, informatics and mother tongue education. All schools are non-vocational schools, with media literacy not being part of their curricula. Teachers in Sweden (*N* = 9) were also teachers in upper-secondary schools from different schools in Uppsala, teaching civics, history, geography, religious studies and informatics. The range of schools has many students with multicultural backgrounds; moreover, students come from a mix of families, with highly educated middle class parents as well as parents with very little education. Teachers in France (*N* = 9) were all working in different school documentation centres in Toulouse and its outer region, as librarians in France are entrusted with media literacy together with partner teachers in history, technology and languages. In Spain (*N* = 7), teachers come from different university departments in Madrid, training teachers in critical pedagogies, information technology in education, communication, digital culture, public relations and information science. Overall, our sample consisted of 12 men and 22 women. Thus, the range of perspectives stretches from in-service secondary and upper-secondary classrooms in France, Sweden and Romania to initial teacher training centres in Spain. Thus, our sample is limited to a small sample of teachers, but still provides insights into the views of key players in the fight against disinformation in different cultural contexts.

### Focus-group interviews and data collection

To understand teachers’ views and understanding of the role of digital tools in developing media literacy aptitudes for their students, we used focus-group interviews. Focus groups have been proven to be an effective method to explore ‘people’s experiences, opinions, wishes and concerns’, while allowing them to ‘pursue their own priorities, in their own terms, in their own vocabulary’ (Barbour and Kitzinger 1999). In the MIL field, this method is embraced more and more as an in-depth method of investigation, as ‘there is a strong trend towards increasingly in-depth qualitative methods’ (Livingstone et al. 2005). One specific feature of focus groups is that it helps to reveal a group consensus, where it exists, while at the same time offering insights into not only what participants think, but also why they think in that particular way (Cyr 2016). However, sometimes consensus might be formed because it is socially desirable; therefore, the experience of the researcher conducting the focus-group is key in addressing the pitfalls of the method.

Additionally, working with artefacts in interviews may elicit important perspectives, especially in a context where materials and methods for teaching are considered (Barton 2015). To stimulate conversation and get direct feedback, we had teachers use and discuss the digital tool designed for fact-checkers, InVID-WeVerify. In the focus-group interviews, we used a fixed set of questions across all groups. This semi-structured approach made it possible for each group to discuss questions about news consumption, fake news, fact-checking and testing of the InVID-WeVerify tool as part of the conversation while also inquiring about their perception of MIL and their experiences with MIL in their institutions. Questions were designed to get teachers’ perspectives on the surrounding society, the arena of formulations and, in particular, map out a spectrum of views from the arena of realisations.

The focus groups took place in February 2020, a neutral moment in the year just before the COVID-19 extreme infodemic. They met in educational settings and lasted between 66 and 90 min. Conversations were directed by experienced researchers and recorded by digital audio recorders. All participation was voluntary and participants gave written consent about their willingness to participate as anonymous respondents, in line with ethical recommendations of each country.

Data from focus groups were analysed using a thematic analysis approach. An English version of the main themes was developed after all interviews were conducted in the four countries and a first familiarisation with the content step was performed. Minimal additional codes were generated during analysis.

## Results

### Teachers’ news consumption in a society of disinformation

The teachers in four different countries overall showed awareness of their own news consumption patterns as well as those of their students and a lucid perception of the incidence of the ‘fake news’ phenomenon and its possible effects. Teachers from all countries claimed to be interested in following news on various media outlets, with Spanish and Swedish teachers specifically mentioning this being very important to them, stating, for instance, how ‘it would be embarrassing as a social studies teacher not to follow the news every day’ (Sweden). Romanian teachers were less interested in general, with some ‘confessing’ that they do not follow news on a daily basis. A teacher claiming not to follow the news regularly stated that ‘I like very much Costi Rogozanu [Romanian online opinion leader, occasionally journalist]; otherwise I think that live discussions with people that you trust are much more constructive and could anchor us much closer to the truth’. However, the Romanian teachers also, spontaneously, mentioned that following professional news (that is, news from the education field) was important. Swedish teachers found the debate on schools in the news very ignorant and ‘never reality-based’ and this statement was supported by other teachers in the focus group.

As far as the type of media outlets, there are some notable differences between and within the four contexts. Romanian teachers followed traditional outlets, TV being their first choice, followed by online sources, rarely social media, with patterns of incidental news exposure or ‘news-find-me’ phenomenon, for instance claiming that ‘I am interested in the official news we often receive directly […]’. Somewhat similarly, teachers from Spain preferred more ‘traditional’ media, mainly radio and online press, and very rarely social media, stating for example that ‘I hear little radio… the main source Internet, but the traditional sources, (…) of social networks nothing’. In Sweden, teachers also consumed traditional and well-established print media, in combination with public service radio, TV and online press, but also mentioned podcasts as important. One Swedish teacher, for instance, described how ‘I start the morning, walking the dog listening to public service radio pod … then local newspaper and then DN [the main national morning newspaper] in the afternoon I listen to news debates on public service radio’. Podcasts from traditional media outlets were perceived as important for teachers in France. In contrast to teachers in other countries, the French teachers also claimed to follow news and updates on social media (Twitter, Facebook, Instagram, but also Snapchat and YouTube for younger ones): ‘Facebook offers a very wide range of accounts. I also follow media that don’t attract me but have interesting themes’. To keep up with the habits of the students, they added this to the many traditional outlets that they followed for more personal reasons. As a particularity for this country, none of the interviewees watched news on TV.

### Teachers’ views on mis- and disinformation

In their understanding of the ‘fake news’ concept, informants from all countries agreed that this is difficult to define, as ‘the vocabulary shifts even without us noticing it’ (France). Some mentioned various avatars of ‘fake news’, such as ‘partially invented news’, out of context news, distorted information (Romania), ‘camouflaging informational realities’, and ‘falsehood and manipulation’ (Spain). Teachers from Romania, Spain and Sweden discussed intentions behind news as important in delimiting concepts. Spaniards and Swedes considered that only news constructed with a clear intention to deceive could fall under the umbrella of ‘fake news’: ‘when we talk about news or false information we are obviously talking about information that has an interest’ (Spain); ‘Fake news is strategical and deliberate with a hidden intention. The publisher knows it is false, this is true for disinformation. Misinformation feels more random and accidental based on carelessness or ignorance’ (Sweden). Romanians, on the other hand, perceived both misinformation and disinformation as ‘fake news’, as ‘both [mis- and dis- information] are fake news. Nobody knows anymore what the intention was’ (Romania).

In Romania and Spain, ‘fake news’ was spontaneously linked by the respondents to the idea of manipulation. Biased news was rather considered a form of misinformation in Sweden, ‘you only see one angle, it goes too fast or you come in without proper background information [or] you may not have the correct filter to be able to read since you have an incorrect road map. In this case, it doesn’t have to be strategic’ (Sweden), but hyper-partisan news was only mentioned as a form of fake news in Romania: ‘It is fake news, as it distorts information to make it advantageous for someone’ (Romania). However, during the discussion, respondents concluded that hyper-partisan news should not ultimately be considered ‘fake news’ because there would be no more ‘normal news’ in this case, referring to the highly polarised media environment in Romania. Parody and satire as types of fake news were also mentioned as possible forms of ‘fake news’ in Romania, but only if not perceived as intended by the audience. Otherwise, parody and satire were not seen as ‘fake news’ but rather associated with it (Tandoc Jr et al. 2018; Nielsen and Graves 2017).

Concerning the prevalence of fake news in society, respondents from France and Romania considered its incidence as ‘very high’, with Romanians offering estimates of 80% of all news, and superlative expressions from French teachers (‘it is so enormous in my institution that I don’t pay attention outside anymore’). In Spain, respondents said they are ‘indeed exposed to false news every day’. Responses from Sweden were more nuanced, with the teachers considering that (accidental) misinformation is more common (especially due to the pressure of time), disinformation not so much. Additionally, they considered that people are more aware of the phenomenon; therefore, new phenomena such as troll factories, for example, are more and more reported nowadays.

The effects of the ‘fake news’ phenomenon were discussed, on both the personal and society level. People from Romania mostly focused on the personal level, suggesting implications for political action among students ‘I have a student who participates in all public protests; he got beaten by the police, because he thought… I tried to explain things, that the truth is always in between, but my explanations were in vain, as he kept going to all public gatherings’. On a personal level, they also mentioned problems with anti-vaccination ‘all the stories about vaccination… I thought these stories were the most dangerous ones for the population’ (Romania). Also at personal level, the French teachers discussed a general feeling of anxiety about the incapacity to escape the phenomenon: ‘they create a breach for fear as in the case of rumour’; ‘there is an omnipresence of this anguish, no means to escape it’ (France). Spaniards were mostly concerned with effects on reputation at both the individual and societal level, considering that fake news affects reputation of ‘individuals, groups, or even the media’ (Spain), while Swedes talked about altering personal or social world views, especially related to events reported in news: ‘Hard to get a fact based world view. Not fake news but biases and sections of focus is a great problem, perhaps greater than fake news’ (Sweden).

### Teachers’ views on MIL and ways to fight disinformation

Regarding ways to fight disinformation, interviewees from Romania and Spain mentioned the importance of media education, and a Spanish teacher stated for instance ‘educate a little in communication, media education. This is the best tool’, echoed by the role of developing critical thinking in France and Spain, and better journalism in Spain and Sweden finding for instance that ‘journalists may self-correct more’. Additionally, legislative measures were seen as a possible solution in Romania (noted as ‘the key is the legislation and its enforcement’), and developing an active state of vigilance by raising awareness was set forth as a solution in France.

Teachers from all countries also highlighted the need to improve MIL, for both teachers and students. French and Spanish teachers are especially concerned about this issue. Spanish teachers identified the lack of visual and audio-visual literacy in education in schools as a central problem in a society plagued by multimodal disinformation. French teachers, in particular, criticised the role of the administration in this lack of training, and were also concerned about the lack of awareness of the need to use digital tools at school, for instance, stating that ‘When I ask for tools, I am told that they are secondary, that there is no time’. French teachers perceived this as an obstacle to supporting students’ search and retrieval of information. Furthermore, they emphasised that ‘media education is not well dispensed’, which hinders adequate training in this area by teachers (France).

Romanian teachers affirmed that a digital tool can be useful in some contexts, but what is more important is the development of critical thinking skills, claiming for instance that ‘If the public does not develop critical thinking skills to make sense of the situation […] I don’t know if it qualifies as fake news, because then it means we don’t have real news anymore’. Without the ability to question the information received, no tool will be useful. In the Swedish context, where teachers found disinformation to be less of a problem, the importance of MIL was discussed primarily as a matter of critical thinking and of students not following the news as a habit. Teachers expressed concerns that young people do not learn from their parents to follow the news anymore, making them an easy target of misinformation, noting how in addition ‘we all need to be better critical thinkers … this is central to our students, don’t trust everything!’.

### Teachers’ views on INVID as a fact-checking tool in education

#### Usefulness

In terms of tool perception, InVID-WeVerify was considered by teachers in all countries as a useful artefact for professionals, but not necessarily for the general public or youth in schools, stating for instance that ‘I don't know if for the general public this type of tool contributes a lot or if they will even use it […] In the end, if he is prepared and educated he doesn’t need the tool and the one who is not, is not served by the tool’ (France). Spanish teachers stated that InVID-WeVerify may be useful for professionals in education, communication and journalism noting that ‘in the journalistic exercise it allows you specific audio-visual analysis, both in video and image’, and the Romanian teachers noted that InVID-WeVerify can be used in different professional fields: ‘politics, journalism, but also anthropologists, psychologists, teachers and statisticians’ may find it useful.

Participating teachers had little or no previous experience from using digital fact-checking tools. Prior experience from using fact-checking tools was entirely absent in Romania, very limited in Sweden (one teacher mentions TinEye), and in Spain (one teacher mentions Google reverse image search) and somewhat limited in France (where some teachers mention TinEye and fact-checking sites). French teachers found a number of difficulties in using the tool at the junior educational level: ‘I don’t see myself doing it at my junior high’. It is a tool that requires initial training before being able to use it in schools: ‘It would be necessary to have media education … Establish a real continuity along the curriculum’ (France). Swedish teachers stated that clearer guidance is needed to adapt the tool to teaching before it can be used in this field. With the current design, Swedish teachers found that it may be used as an artefact highlighting how professional journalists today have to work very hard to detect fake news: ‘I find this tool to be a nice digital environment to show students the complexity that journalists face today to fact-check information. Just letting students see a tool like this may be good for many students [to see that] this is for real!’

When discussing the usefulness of InVID-WeVerify, Romanian teachers focused on how it could help their students to fact check misleading information, and also as a means to help young people cope with depression: ‘I think of the young girls, and their depression caused by Instagram pictures. Seeing all the pictures currently promoted on Instagram, all young girls dream of looking like that. If they find out that it was not real, that the picture […] was altered in Photoshop, that they starved for nothing. […] Clearly, the tool has features related to images and videos, and it is clearly helpful in these kinds of contexts’ (Romania). Swedish teachers also discussed usefulness from the students’ point of view and found the tool to be ‘interesting but hard to understand’, underscoring how ‘students will find this super hard to use’. Teachers in Sweden stated that if students are to be able to use it in the current design, then ‘we as teachers need to show students this is what you can do’.

The Spanish teachers stated that they would only use it for specific cases, but not to obtain information on a daily basis, stating for instance that ‘for the normal user I see it as difficult, or he need to be is very interested’. In contrast, French, Romanian and Swedish teachers showed interest in using the tool in their future teaching, provided some improvements are incorporated. Romanian teachers found it ‘too complicated, but would [try to] use it’ in the classroom despite the complexity of the tool. Teachers in Romania found that InVID-WeVerify in its current design should preferably be introduced by an expert, which may also ‘matter a lot for [students] motivation to use it’. Among French teachers, the tool was also perceived to be ‘a good tool for popular education associations and for the police’. French, Romanian and Swedish teachers highlighted the need to match the tool to content in schools and disciplines. Romanian teachers also proposed developing an app for students to use. Swedish teachers, positive to trying the tool in classrooms, called for more scaffolds for teachers and students. One teacher stated that ‘I would personally need more instructions like: 1 do this, 2 do this, 3 do this’, and other teachers called for more simple instructions and examples for students. It could also be useful to fact-check students’ biased news from YouTube as well as when they come into the classroom with biased or false news from areas of conflict’ (Sweden).

#### Adapting the tool to the arena of realisations

In relation to the improvements that the tool would require to be useful in classrooms, the French, Romanian, Spanish and Swedish teachers commented both on the limitations of InVID-WeVerify for analysing texts, and on the information that the tool returns once the news analysis has been carried out. Some aspects that could be added are a glossary, a discussion box (chatbot), error messages and discussions with the developers of the plug-in, and the inclusion of a soundtrack in the videos (France). Romanian teachers suggested that it could be developed into a mobile app ‘[…] because, you know, the kids, they are always on their phones, and rarely in front of a computer. If it were available on smartphone, then it might get interesting; they might be tempted to click and click, and to discover more’ (Romania). Furthermore, they all emphasise that a much larger database of examples should be added, since having ‘more practice examples would help them [the kids] to be more thorough, more organised, to value quality’ (Romania). This is also highlighted by a teacher in Sweden who found ‘interactive [one of the sections of the plug-in] to be useful with examples. This was interesting and also instructive with fixed examples to engage with’. Along these lines, another teacher stated that ‘if you give students a set of carefully selected images to do “forensic” [one of InVID-WeVerify’s functionalities] on, they should be able to use the tool for this and make some sense out of the analysis, but just going to ordinary current news seems too difficult. Ambiguity is a great challenge in non-curated digital news feeds. It would, of course, be great to have students dig into the most current but this is hard; they may end up with nothing if they do the search themselves’.

In sum, teachers from different countries agreed that the tool should be improved to facilitate its use by improving its usability, providing ready-checked examples, making changes that facilitate its use by young people and providing guidelines for adapting the tool to teaching. Difficulties with navigation menus were highlighted in all contexts, as well as limitations for text analysis, in contrast to the utility for analysing images. In a nutshell, an artefact like InVID-WeVerify was perceived as interesting for cognitive support, but it should provide more intuitive technical and navigation features to be more useful in education.

## Discussion

The European arena of formulations is relatively favourable to media education and fact checking with the European Commission Plan of Action against disinformation underscoring the importance of MIL to safeguard citizens (European Commission 2018) and the AVMS Directive making Media Education a member state obligation. When we go to the arena of realisations, we find that ideas formulated in international ideological curricula (Goodlad 1979) come with diverse challenges of implementation in different contexts across Europe. When we investigate how a digital tool may be implemented in classrooms (RQ4) in the light of teachers’ patterns of news consumption (RQ1), understanding of the term ‘fake news’ (RQ2) and their views on measures against disinformation (RQ3), we find that teachers in France, Romania, Spain and Sweden live in societies with very different access to news, diverse patterns of news consumption and presence of ‘fake news’. We also find that digital educational resources differ across borders. Promoting transliteracy in schools therefore holds specific challenges, but also more general challenges of disinformation. Even if teachers in all four countries welcome digital tools like InVID-WeVerify to stimulate MIL in classrooms, they find it difficult to implement in the classrooms. Below we discuss this complexity and possible ways forward.

### MIL in different European contexts: formulations disconnected from realisations

Teachers’ views, stemming from the arena of realisations, reflect elements of Hallin and Mancini’s model, as revisited by Matović, Juraitė and Gutiérrez for MIL. It is evident that teachers live in countries with more or less support when educating students to become critical thinkers who are able to navigate in new digital environments. In our sample, Spain is part of the polarised pluralist model as is France (though the latter is borderline with the democratic corporatist model), Sweden is part of the democratic corporatist model and Romania pertains to the hybrid model. Sweden and Romania appear to be at the two extreme poles, in terms of realisations, while France and Spain offer mitigated situations.

Perspectives of Romanian teachers highlight how the media situation makes it difficult for them and their students to follow credible news. They also identify how they have little resources to support students’ media literacy. This is in sharp contrast to the arena of realisations in Sweden, where teachers find the media situation in society to be manageable and in education, they find a lot of support in the national curricula for MIL. In Sweden, most students also have one-to-one computers, making the technological situation better than in many other countries. This contrast highlights how students with some of the greatest challenges of disinformation may be provided with the least support from education to counter this, while the opposite seems to be true for Swedish students. However, this does not mean that Swedish students are skilled at navigating digital news. Previous research actually point to the opposite. Especially, over-confident students in Sweden may struggle to separate credible news from disinformation (Nygren and Guath 2019). Nonetheless, the educational challenge seems far greater in the arena of realisations in Romania than in Sweden.

The development of MIL in France and Spain seem to hold more possibilities than in Romania but less than in Sweden. This reflects the polarised pluralist model, where the level of professionalisation of teachers is key to their sense of competence to address the issue of disinformation, and the degree of state intervention is expected to play an important role for guidelines and tool provision. Furthermore, these teachers see media literacy as an activity that can be shared outside schools. This can explain why they feel limited in their realisations because of lack of support from their authorities and from lack of initial or continuous training in using digital tools in education. Consequently, they are not over-confident in their ability to deal with disinformation but paradoxically show a keen interest in self-training and keeping abreast of students’ uses and practices, as if to compensate, at their level of realisations, the gaps they feel in their educational system.

Teachers also express concerns about a social and digital divide between students with different backgrounds. To take on this challenge, teachers in France have started following news in the social media outlets where students may find information and disinformation. They see great challenges and call for more support, especially for students with poor socio-economic backgrounds. Swedish teachers see a similar challenge today, when their students do not live with parents who read the morning newspaper, passing on constructive news habits to the younger generation. Teachers note how the digital media has made some productive habits less visible, and schools need to make it evident where and how to find reliable news.

The lack of presence of MIL in the school curricula is bemoaned, as is the lack of teacher training in these areas, not least when it comes to visual and audio-visual information. This is especially true for teachers in contexts where teachers see disinformation as a problem not addressed in the curricula. As for the criteria by Matovic et al. (2017), they are confirmed: the level of professionalisation of teachers is perceived as needing much improvement. Teachers note the role of parents and the degree of state intervention as problematic. Especially teachers in Rumania seem to lack resources and support in the curricula. They also seem to be more restricted by national guidelines, making it more difficult to add important dimensions of MIL in their teaching, when they find this necessary. In contrast, Swedish teachers seem to have more freedom to decide what to do in their classrooms and be critical towards the educational policies. Among French and Spanish teachers, they offer many perspectives on MIL activities in the arena of realisations, but they also express that they often see a lack of support from the arena of formulations. French teachers were the most explicit, in adopting a critical position when making their comments towards the educational administration—indicating that they lack some of the support that Swedish teachers may have and also that they may have more room than Romanian teachers to be critical towards the state.

In the light of these results, the promotion of MIL to counter disinformation highlighted in formulations by the European Commission (2018) seems to be disconnected from the arena of realisations, especially in Rumania. ‘Improving citizens’ media literacy to understand how to spot and fend off disinformation’ (European Commission 2018) will need more support, with a nuanced consideration about the different challenges teachers may face in different countries and contexts.

### A professional tool in education to promote technocognition and transliteracy against disinformation

The technocognition of teachers regarding fact checking in digital environments did not match well with the tool designed for and used by professional fact-checkers. Without the knowledge and skills of fact-checkers, they struggled to use it as well as to see the purpose of what they find out when using it. As noted by Lewandowsky et al. (2017), technology and cognition need to go hand in hand to counter disinformation. However, just handing the latest technology to teachers and students may not be productive in this way, which is why transliteracy suggests a MIL scaffolded design. Across borders, teachers viewed the tool as difficult to implement in its current design in education. Teachers pointed out several challenges when using a tool like InVID-WeVerify in the arena of realisations: students’ and teachers’ lack of training in MIL, technical difficulty of the tool or lack of interest on the part of public administrations or the teachers themselves.

Teachers’ perspectives on using InVID-WeVerify highlight how implementation is possible and may trigger interest among students (Sun et al. 2018). Nevertheless, teachers and students seem to need hard and soft scaffolds and updated heuristics to be able to use such artefacts in constructive ways, as also noted in previous research (Nygren and Vikström 2013; Nygren et al. 2014; Saye and Brush 2004). Teachers find this new technology to be complicated and without much added value unless it is updated to better fit into the context of teaching and learning about disinformation.

This view needs to be considered in the light of their previous lack of experience from using fact-checking tools and also in the light of their relative lack of transliteracy competences, especially in the converging threads of visual media literacy and data literacy. Teachers note that using a tool like InVID-WeVerify comes with issues of distraction, time-consuming technical problems, lack of subject specific knowledge and added stress from technology. What the teachers underscore as challenges are in line with previous research on digital tools in education (e.g. Kirschner and De Bruyckere 2017; Nygren and Vikström 2013).

Teachers also note the potential of fact-checking images and videos in updated ways that point to an appetite in updating their competences and knowledge in MIL. Using a tool like InVID-WeVerify in teaching seems to hold great potential if the tool can be redesigned to better fit the technocognition and the transliteracy competences of diverse users. Greater usability would ask less from teachers’ skills and digital expertise when using it in practice. The complexity of the tool can also be levelled out in practice by better MIL among teachers and students, with InVID-WeVerify being embedded in civics education and sense-making practices for digital citizenship. This should be addressed in future design cycles with classroom interventions, which will provide lesson plans on disinformation at large, with InVID-WeVerify as one of the featured tools for visual literacy. Congruent with Akkerman et al. (2013), we confirm that dialogue with teachers about technology is essential in educational design research. Insights from this study were a solid basis for an educational design that yielded noteworthy results from using the digital tool in classrooms. Insights from teachers helped us design a cross-national intervention with significant effects on students' attitudes and abilities to fact-check disinformation (Nygren et al. 2021).

It is evident that in order to face the social disruption of disinformation, we need to consider multiple challenges in diverse contexts. Teachers identify important disconnects between the arena of formulations about how education should counter disinformation and what is possible in the arena of realisations. This may be a hindrance to building resilience to “fake news” at early stages, among young citizens-to-be. Digital tools for fact-checking, today lacking in education in all four countries, may be constructive to use, if implemented with careful considerations about technocognition and in correlation with transliteracy. However, the many critical and complex aspects identified in our focus groups underscore how it is very hard for educators to live up to the high hopes about education against disinformation formulated by international organisations. We also find that teachers themselves may have little or no experience from using fact-checking tools. They describe the need to improve media literacy by (a) improving the practical ability in the use of the digital fact-checking tool and (b) reinforcing the ability to think critically. A digital tool popular among professional fact-checkers may be perceived as useful or very difficult to use in education, depending on the design, context and previous knowledge among teachers but it yields the promise of fruitful exchanges across those two communities of practice and interest.

## Supplementary Information

Below is the link to the electronic supplementary material.

Supplementary file1 (DOCX 14 kb)

Supplementary file2 (DOCX 13 kb)

## Data Availability

See Appendix 1 and 2 for protocol for recruitment and guidelines for focus-group interview.
