# Supplementary material for: Teachers’ views on disinformation and media literacy supported by a tool designed for professional fact-checkers: perspectives from France, Romania, Spain and Sweden
Source: SN Soc Sci. 2022 Apr 9;2(4):40. doi: 10.1007/s43545-022-00340-9 (PMC8994523; doi:10.1007/s43545-022-00340-9)
Supplement: Supplementary file 1 — Supplementary file1 (DOCX 14 kb) [file 43545_2022_340_MOESM1_ESM.docx]

Appendix 1 Guide for focus interviews

**FOCUS-GROUP guide for InVID-WeVerify**

**Intro**:

*General background about the project*

*Focus group procedure: rules, anonymity, data processing, etc.*

**Introduce each other**:

- Name, profession, areas of interest (distribute labels or name tags)

1. **General background**
   1. We would like to invite you to talk about how and from where you get your daily news these days

- Media outlets
- Interest in political and social news
- Incidental news exposure
- News avoidance
  1. There is a lot of talk lately in the media about the “fake news” phenomenon, online disinformation, computational propaganda, etc. We would like to invite you to discuss together these phenomena.
- Definitions (what they mean by “fake news”; only after spontaneous discussion over, ask about: intention to deceive, facticity, financial gains)
- Perceived frequency / prevalence
- Danger / harm; social significance; importance of possible (negative?) effects
- Measures to combat (discuss fact-checkers, media literacy, tools, etc)
  1. We have discussed about the fact-checking companies and various tools used nowadays to combat “fake news” and online disinformation. We would like to know to what extent have you ever used any of these in the past.
- Explore knowledge of fact checkers and fact checking tools
- Explore professional experience, if any
- Explore people’s criteria for identifying fake news (if no tools used, or additionally): ask (only after spontaneous discussion) about: journalists’ reputation, media outlets’ reputation, checking with friends
- Explore people’s interest in tools such as InVID-WeVerify

**InVID-WeVerify plugin**: Invite participants to use it

1. **InVID-WeVerify feed-back**
   1. You had the chance to use and play a little with the InVID-WeVerify tool now. Let’s talk about the features of this tool.

- Tutorials, Classroom and Interactive, explore: if and how much they used these, usefulness, appeal, novelty
- Features on the left side: general discussion: clarity, usefulness, easiness of use
- Features on the left side: ask about the ones that people mention spontaneously
- Ask about the most useful, the least useful
  1. Moving to a different topic, let’s discuss now to what extent do you think this could be useful to you and others at a professional and personal level
- Explore perceived professional usefulness
- Explore personal use in news consumption habits
  1. You all have now a general idea about this tool. Do you think you will use this tool professionally in the future?
- Explore reasons why (yes and no): time, usefulness, easiness of use
- Explore possible use of similar tools
- Explore use of this tool, with changes: what kind of changes
- Would you recommend the tool to friends or interested people? Why?

**Closure**

We covered topics related to various aspects, such as fake news, online disinformation, fact-checkers, toolkits, plugins to debunk false information, etc. Do you think there is anything else that you consider important and we should discuss before leaving, today?

*Thank the participants for their cooperation and insightful comments.*
