# Supplementary material for: Teachers’ views on disinformation and media literacy supported by a tool designed for professional fact-checkers: perspectives from France, Romania, Spain and Sweden
Source: SN Soc Sci. 2022 Apr 9;2(4):40. doi: 10.1007/s43545-022-00340-9 (PMC8994523; doi:10.1007/s43545-022-00340-9)
Supplement: Supplementary file 2 — Supplementary file2 (DOCX 13 kb) [file 43545_2022_340_MOESM2_ESM.docx]

Appendix 2. Focus-groups recruitment protocol in each country

**Two ways of recruitment**

1. People for focus groups with teachers will be recruited from the beta-testers (should tell them about the focus groups from the beginning). This way, we make sure they played with InVID before the focus-groups.
2. Alternatively, teachers could be recruited within the general protocol of classrooms interventions, to make sure they used the toolkit before the focus groups.

Back-up plan for teachers who have not used the toolkit prior to the focus groups: he alternative way is to have new people and to present them the tool after the first half of the focus-groups, and give them 15-20 mins to use it. This means that we should find a way to organize the focus groups in a room with individual access to computers on which the page where they could download the plugin should be opened: <https://www.invid-project.eu/tools-and-services/invid-verification-plugin/> The focus groups will continue after the 15-20 mins.

**Profile of the teachers**

1. Six to 12 (ideally 8) secondary school teachers from urban areas
2. Teaching classes of social sciences and humanities (ideally), but could teach also any class that could accommodate media literacy intervention with the InVID-WeVerify toolkit
3. Should have been teaching for at least 3 years
4. Should not know each other
5. Should not know the moderator
